# Renal capsule patient-derived xenograft model for gastric cancer: establishment and MRI characterization

**DOI:** 10.3389/fimmu.2025.1683916

**Published:** 2025-11-05

**Authors:** Zhenyu Yin, Qian Liu, Ewetse Paul Maswikiti, Zhuanfang Wang, Yuping Bai, Lin Xiang, Yuhan Wang, Bin Ma, Lei Gao, Jianming Shi, Hao Chen

**Affiliations:** ^1^ The Second Hospital and Clinical Medical School, Lanzhou University, Lanzhou, China; ^2^ Humanized Animal Model Laboratory, Lanzhou University Second Hospital, Lanzhou, China; ^3^ Surgical Oncology Department, Lanzhou University Second Hospital, Lanzhou, China; ^4^ The Key Laboratory for Digestive Tumors, Lanzhou University Second Hospital, Lanzhou, China

**Keywords:** renal capsule, gastric cancer, patient-derived xenograft, establishment, MRI characterization

## Abstract

**Objective:**

Identify factors associated with the engraftment of gastric cancer patient-derived xenograft (GC PDX) in the renal capsule and explore optimal MRI sequence parameters for observing renal capsule PDX.

**Methods:**

Tumor tissues from 33 gastric cancer patients were cut into fragments of 1×1×1 mm, 2×2×2 mm, and 3×3×3 mm, then transplanted beneath the renal capsule of NOD/SCID mice within 2, 8 and 24 hours. Depending on tissue availability, tumor samples from each patient were implanted into 1–4 mice, totaling 73 mice. Clinical data were collected. Tumor growth was monitored weekly via MRI. T1WI, contrast-enhanced T1WI, T2WI was used to measure tumor length. After euthanasia (10g/L sodium pentobarbital, 180mg/kg, intraperitoneal), tumors were excised, and caliper-measured were compared with MRI results. The xenografts were serially passage into new mice for three generations. Histopathological (H&E), Ki67 immunohistochemistry were performed to assess similarity with primary tumors.

**Results:**

Tumors from 20 out of 33 patients successfully engrafted in 28 out of 73 mice. The 2×2×2 mm grafts and transplantation in 2 hours had a higher success rate. Patient serum albumin was associated with successful engraftment. PDX exhibited isointense and hyperintense on T2WI and marked enhancement on T1WI post-contrast. No significant difference was observed between MRI and caliper-measured. H&E staining, Ki67 expression confirmed that PDX tumors retained histological features of primary tumors.

**Conclusion:**

Optimal conditions for establishing GC PDX models involve transplanting 2×2×2 mm tumor fragments within 2 hours. MRI enables sensitive tumor detection and accurate size quantification. T2WI was the most effective and efficient imaging technique. Providing an efficient preclinical model for personalized therapy of gastric cancer.

## 1 Introduction

Tumors pose a great threat to human health, and GC is the fifth most common cancer worldwide and the fourth leading cause of death. According to global cancer statistics, 2020 (1), there were approximately 1.08 million new cases of GC and 760,000 deaths worldwide (2, 3).

Although chemotherapy, targeted therapy and immunotherapy drugs for gastric cancer have been widely used in clinical patients, not all patients can benefit from them. A clinical trial demonstrated that the 3-year disease-free survival rates were 51.1% in the adjuvant capecitabine and oxaliplatin group, 56.5% in the adjuvant S-1 and oxaliplatin group, and 59.4% in the perioperative S-1 and oxaliplatin group (4). The progression-free survival of apatinib combined with chemotherapy is 3.72 months, the response rate for apatinib combined with chemotherapy remained low (5). A study on immune checkpoint inhibitors combined with chemotherapy demonstrated that, compared to placebo plus chemotherapy, nivolumab combined with oxaliplatin significantly improved progression-free survival in advanced gastric cancer (10.45 vs. 8.34 months), but did not improve overall survival (17.45 vs. 17.15 months) (6). The reason may be the tumor heterogeneity, therefore for different GC patients, need the different treatment strategies (7).

In order to explore effective personalized treatment methods, researchers have developed the PDX model, compared with the cell line model (8–11), The histological and genomic characteristics of the patient tumor were preserved. These models can replace patients for individualized drug screening, so as to achieve the purpose of precise therapy (12).

NOD/SCID mice were established by crossbreeding non-obese diabetic (NOD) and Server combined immune-deficiency (SCID) mice, which do not develop diabetes because they have lost functional T lymphocytes (13, 14). Additionally, various innate and adaptive immune defects were found in these mice, making them better recipients for human hematopoietic stem cell and solid tumor transplantation, and increasing the success rate for human cell and tissue transplantation (15).

The PDX model holds potential importance for cancer research and clinical translation. However, the success rate of gastric cancer PDX is low (12-39.6%), and the process is time-consuming (16–20). Some studies suggest that renal capsule transplantation may improve the engraftment rate, yet few reports have identified factors associated with the successful establishment of gastric cancer PDX tumors in the renal capsule. Additionally, there is limited research on MRI-based observation of gastric cancer PDX in the renal capsule. Therefore, this study aims to identify factors associated with the engraftment of gastric cancer PDX in the renal capsule and explore optimal MRI sequence parameters for observing renal capsule PDX.

## 2 Materials and methods

### 2.1 Tumor specimens and experimental animals

A total of 33 gastric cancer patients were admitted in the department of surgical oncology, Lanzhou University Second Hospital. All human experiments were approved by Lanzhou University Second Hospital ethics review (2024A-626).

The experimental animal strain was NOD/SCID, male, 4–6 weeks old, weighing 14-20g, purchased from Vital River Laboratory Animal Technology (BeiJing, CN). All animal experiments were approved by the laboratory animal care and ethics committee of Lanzhou University Second Hospital ethics review (D2024-596).

### 2.2 The clinical data of patients

Since the tumor samples for transplantation were obtained from gastric cancer patients, to compare whether factors like pathological type and blood biochemical levels affect engraftment outcomes, we collected clinical patient information including: age, gender, pathological type, differentiation grade, Borrmann classification, Ki-67 positive cell count, AJCC stage, Absolute Neutrophil Count (ANC), Hemoglobin (HGB), Fibrinogen (FIB), Carcinoembryonic Antigen (CEA), Carbohydrate Antigen 125 (CA125), Carbohydrate Antigen 19-9 (CA199), Albumin (ALB).

### 2.3 Establishment of PDX model of gastric cancer

Based on the tumor tissue availability from 33 patients, xenografts were implanted into 1–4 NOD/SCID mice per patient, utilizing a total of 73 mice (**Supplementary Tables S1**, **S2**).

The harvested tumor tissue was rinsed in tissue preservation solution to remove blood and necrotic debris. Based on tissue volume, it was then dissected into 1×1×1 mm, 2×2×2 mm, and 3×3×3 mm fragments using tissue scissors. NOD/SCID mice were anesthetized with 10g/L sodium pentobarbital (60 mg/kg for injection, method of delivery: intraperitoneal. Sigma, USA). The mice were immobilized on the operating platform using rubber bands. The abdominal area was disinfected with iodophor, and hair was removed using a microtome blade. Subsequently, the skin and abdominal wall were sequentially incised with ophthalmic scissors. A retractor was then used to spread the abdominal wall, exposing the kidneys. Tumor fragments were implanted beneath the renal capsule in 2, 8 and 24 hours (ex vivo time, from gastrectomy to beginning of the PDX). The surgical site was disinfected with iodophor, and mice were ear-marked before being returned to their cages for recovery.

### 2.4 Observing PDX with MRI

MRI scan was conducted weekly using MR (3.0T, Philips, Netherlands), protocols details as follows: T1WI transverse plane: TR384ms, TE8.6ms. T2WI transverse plane; TR3000ms, TE80ms, FOV 60mm×60mm, Matrix 200×195, 1.5mm thick layer was used. For contrast-enhanced imaging, tail-vein injection of Gd-based contrast agent (Gadoteric Acid Meglumine Salt Injection, Hengrui CN, volume 30uL, dose 0.1 mmol/kg of body weight). The tumor length and width were measured by T2WI and T1WI+C with syngo fastView (Siemens, Germany), tumor volume = 0.5 × length × width^2^. Observe tumor growth until it reaches 100–500 mm³, euthanize the mice (chemicals: 10g/L sodium pentobarbital, 180mg/kg for injection, method of delivery: intraperitoneal, Sigma, USA), extract the tumors, measure the tumor diameter with a vernier caliper, compare it with the last MRI results, and perform pathological examination on the PDX tumors.

### 2.5 H&E and immunohistochemical staining

Patient tumor specimens and xenografts were fixed in 10% neutral buffered formaldehyde and paraffin-embedded for histological (H&E) and immunohistochemical (IHC) analysis. For H&E staining, 4-μm sections were stained with the H&E solution (Servicebio, CN) according to standard protocols. For IHC staining, antigen retrieval was performed using citrate buffer under heat-induced conditions. The sections were incubated with Ki67 antibody (Servicebio, CN, GB121499-100) at 4°C overnight, followed by incubation with secondary antibody at room temperature for 20 minutes. DAB was used as the chromogen with a 20-second incubation period, followed by counterstaining with hematoxylin. Images were acquired under microscopy, and the percentage of positively stained areas was quantified using ImageJ software. Statistical analysis was performed using Prism software.

### 2.6 Statistical method

Data processing and analysis were performed using Prism and Zstats 1.0 (www.zstats.net). If data follows a normal distribution with homogeneity of variance: Independent samples t-test is recommended. If normally distributed but with unequal variances: Use Welch’s t-test. Non-parametric rank sum test was used for non-normal distribution. χ2 test was used to compare the inter-group rates. 0.05 was considered statistically significant.

## 3 Result

### 3.1 Baseline characteristics

Tumor tissues from 33 gastric cancer patients were transplanted into 73 mice. **Supplementary Table S1** summarizes the patients’ baseline characteristics: mean age was 60.1 years, with 20 males (60.61%). Adenocarcinoma in 30 patients (90.91%), neuroendocrine carcinoma in 2 patients (6.06%), and squamous cell carcinoma in 1 patient (3.03%). Tumor differentiation included moderately differentiated in 11 patients (33.33%), poorly differentiated in 21 patients (63.64%), and well-differentiated in 1 patient (3.03%). Borrmann classification showed type I in 4 patients (12.12%), type II in 12 patients (36.36%), type III in 16 patients (48.48%), and type IV in 1 patient (3.03%). AJCC staging distribution was stage I in 6 patients (18.18%), stage II in 16 patients (48.48%), stage III in 9 patients (27.27%), and stage IV in 2 patients (6.06%).

### 3.2 Establishment of PDX models

The number of mice receiving tumor implants per patient and the successful engraftment counts are summarized in **Supplementary Table S2**. Among the 73 mice:29 (39.73%) received 1×1×1 mm tumor fragments, 28 (38.36%) received 2×2×2 mm fragments, 16 (21.92%) received 3×3×3 mm fragments. For ex vivo timing: 36 mice (49.32%) were grafted in 2 hours (from gastrectomy to beginning of the PDX), 29 mice (39.73%) were grafted in 8 hours, 8 mice (10.96%) were grafted in 24 hours.

### 3.3 Factors related to successful engraftment of PDX

To investigate potential factors associated with PDX tumor engraftment success, we categorized the parameters into patient characteristics and experimental variables.

**Figure 1:**
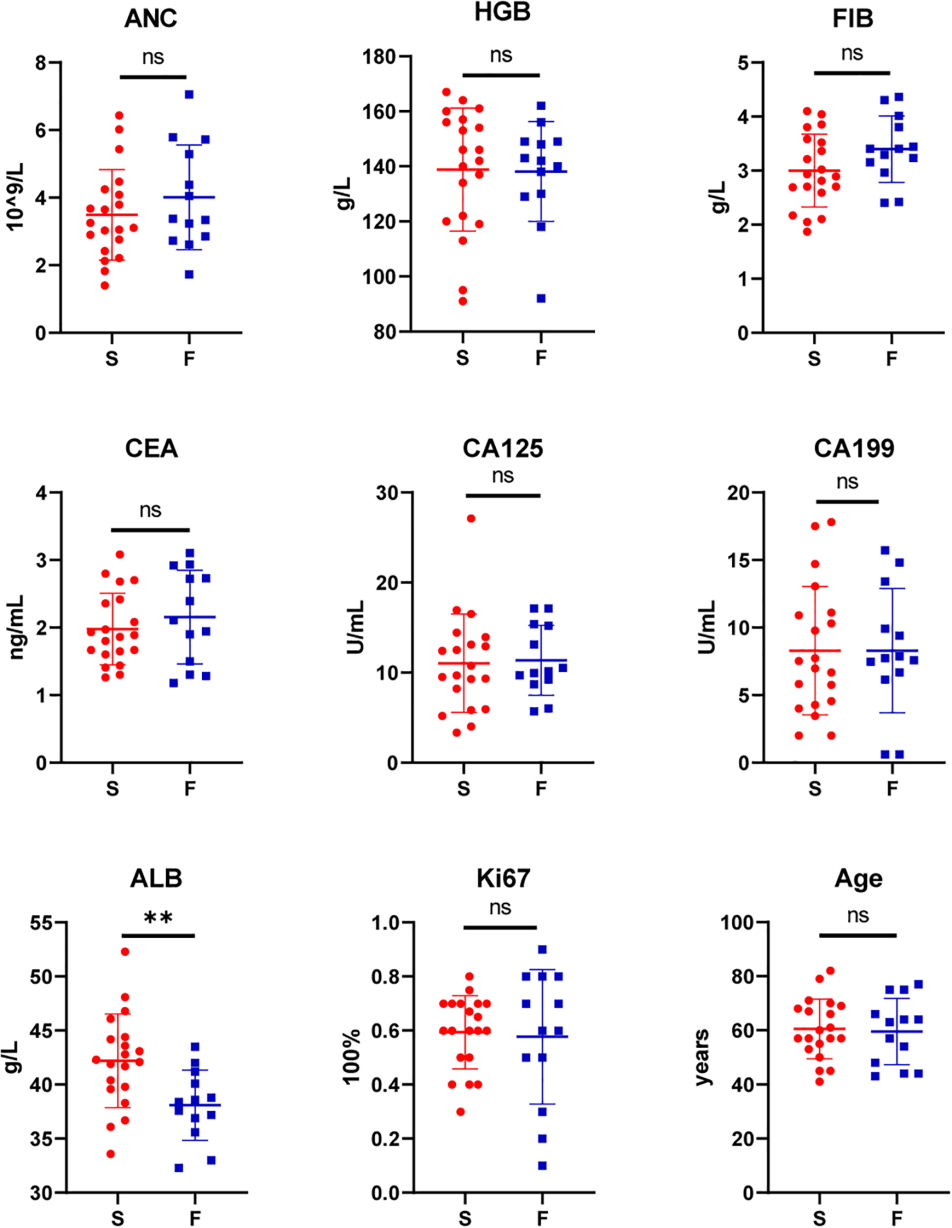
Comparison of patient characteristics between the success (S, n=20) and failure group (F, n=13): Absolute Neutrophil Count (ANC), Hemoglobin (HGB), Fibrinogen (FIB), Carcinoembryonic Antigen (CEA), Carbohydrate Antigen 125 (CA125), Carbohydrate Antigen 19-9 (CA199), Albumin (ALB), Proliferation marker protein Ki-67 (Ki67), Age. (**P<0.01; ns, P>0.05).

Among the 33 patients in this study, engraftment failed in 13 (39.39%) and succeeded in 20 (60.61%). No statistically significant differences were observed between the two groups in terms of gender, Borrmann classification, pathology, degree of differentiation, or AJCC staging (P > 0.05) (**Table 1**). Similarly, there were no significant differences in age, ANC, HGB, FIB, CEA, CA125, CA199 or Ki67-positive cell count (P > 0.05). However, there was a significant difference in albumin levels between the successful group and the failed group [(42.2 ± 4.34) g/L vs (38.09 ± 3.26) g/L, t=2.91, p=0.006] (**Figure 1**). All variables followed a normal distribution (**Supplementary Figure 1**).

**Table 1:** Comparison of patient characteristics between the success and failure group.

Variables	Total (n = 33)	Failed (n = 13)	Successful (n = 20)	Statistic	*P*
Gender, n (%)				–	1.000
female	13 (39.39)	5 (38.46)	8 (40.00)		
male	20 (60.61)	8 (61.54)	12 (60.00)		
Borrmann, n (%)				–	0.351
I	4 (12.12)	1 (7.69)	3 (15.00)		
II	12 (36.36)	3 (23.08)	9 (45.00)		
III	16 (48.48)	8 (61.54)	8 (40.00)		
IV	1 (3.03)	1 (7.69)	0 (0.00)		
Pathology, n (%)				–	0.299
adenocarcinoma	30 (90.91)	12 (92.31)	18 (90.00)		
neuroendocrine	2 (6.06)	0 (0.00)	2 (10.00)		
squamous cell	1 (3.03)	1 (7.69)	0 (0.00)		
Differentiated, n (%)				–	0.176
moderately	11 (33.33)	6 (46.15)	5 (25.00)		
poor	21 (63.64)	6 (46.15)	15 (75.00)		
Well	1 (3.03)	1 (7.69)	0 (0.00)		
AJCC, n (%)				–	0.173
I	6 (18.18)	1 (7.69)	5 (25.00)		
II	16 (48.48)	5 (38.46)	11 (55.00)		
III	9 (27.27)	6 (46.15)	3 (15.00)		
IV	2 (6.06)	1 (7.69)	1 (5.00)		

-Fisher exact.

Regarding experimental parameters (tissue size and ex vivo time), among the 73 mice studied, engraftment failed in 45 (61.64%) and succeeded in 28 (38.36%). Significant differences were observed between the two groups in tissue size and ex vivo time (P < 0.05). In the successful group:16 mice (57.14%) were implanted with tissue of size 2×2×2 mm,8 mice (28.57%) with size 1×1×1 mm, and 4 mice (14.29%) with size 3×3×3 mm. Additionally: 20 mice (71.43%) were implanted in 2 hours, 6 mice (21.43%) were implanted in 8 hours, 2 mice(7.14%) were implanted in 24 hours (**Table 2**).

**Table 2:** Comparison of experimental parameters between the success and failure group.

Variables	Total (n = 73)	Failed (n = 45)	Successful (n = 28)	Statistic	*P*
Graf size(mm), n(%)				χ²=6.81	0.033
1×1×1	29 (39.73)	21 (46.67)	8 (28.57)		
2×2×2	28 (38.36)	12 (26.67)	16 (57.14)		
3×3×3	16 (21.92)	12 (26.67)	4 (14.29)		
Ex vivo time(h), n(%)				–	0.013
2	36 (49.32)	16 (35.56)	20 (71.43)		
8	29 (39.73)	23 (51.11)	6 (21.43)		
24	8 (10.96)	6 (13.33)	2 (7.14)		

χ²Chi-square test, -Fisher exact.

### 3.4 Observing the PDX model with MRI

**Figure 2:**
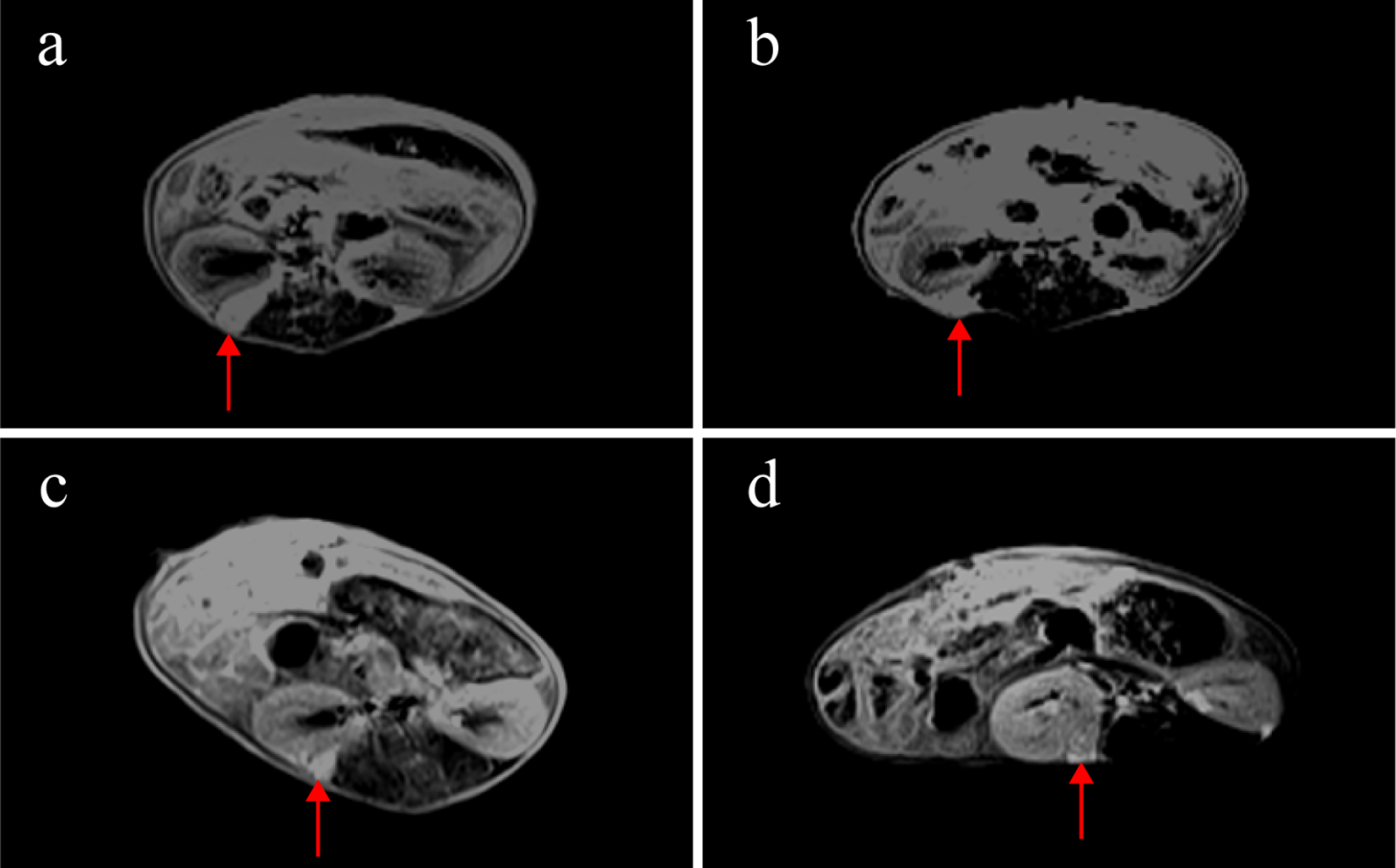
MRI observations of a PDX model in renal capsule (arrow). **(a)** on the 8th day after establishment, the length of the tumor was 2.5mm, T1WI+C: **(b)** on the 16th day, the length of the tumor was 2.1mm, T1WI+C; **(c)** on the 25th day, the length of the tumor was 2.0mm, T1WI+C; **(d)** on the 32nd day, the length of the tumor was 2.7mm, T1WI+C; T1-weighted contrast-enhanced imaging (T1WI+C).

**Figure 3:**
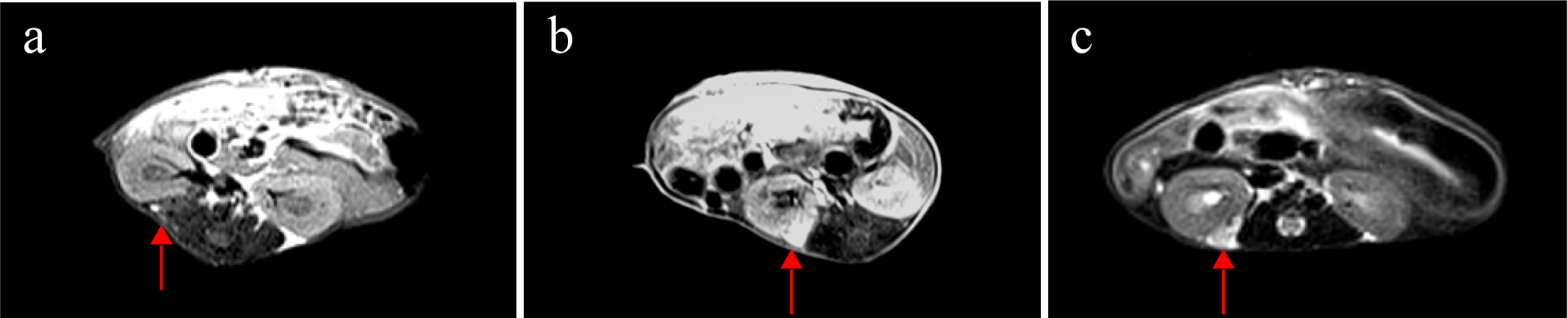
MRI findings of renal capsule PDX in T1WI, T1WI+C, and T2WI sequences(arrow). **(a)** Isointense and Hypointense on T1WI. **(b)** marked enhancement on T1WI contrast-enhanced(T1WI+C) scans. **(c)** Isointense and Hyperintense on T2WI, companying with renal compression. T1-weighted imaging (T1WI), T1-weighted contrast-enhanced imaging (T1WI+C), T2-weighted imaging (T2WI).

**Figure 4:**
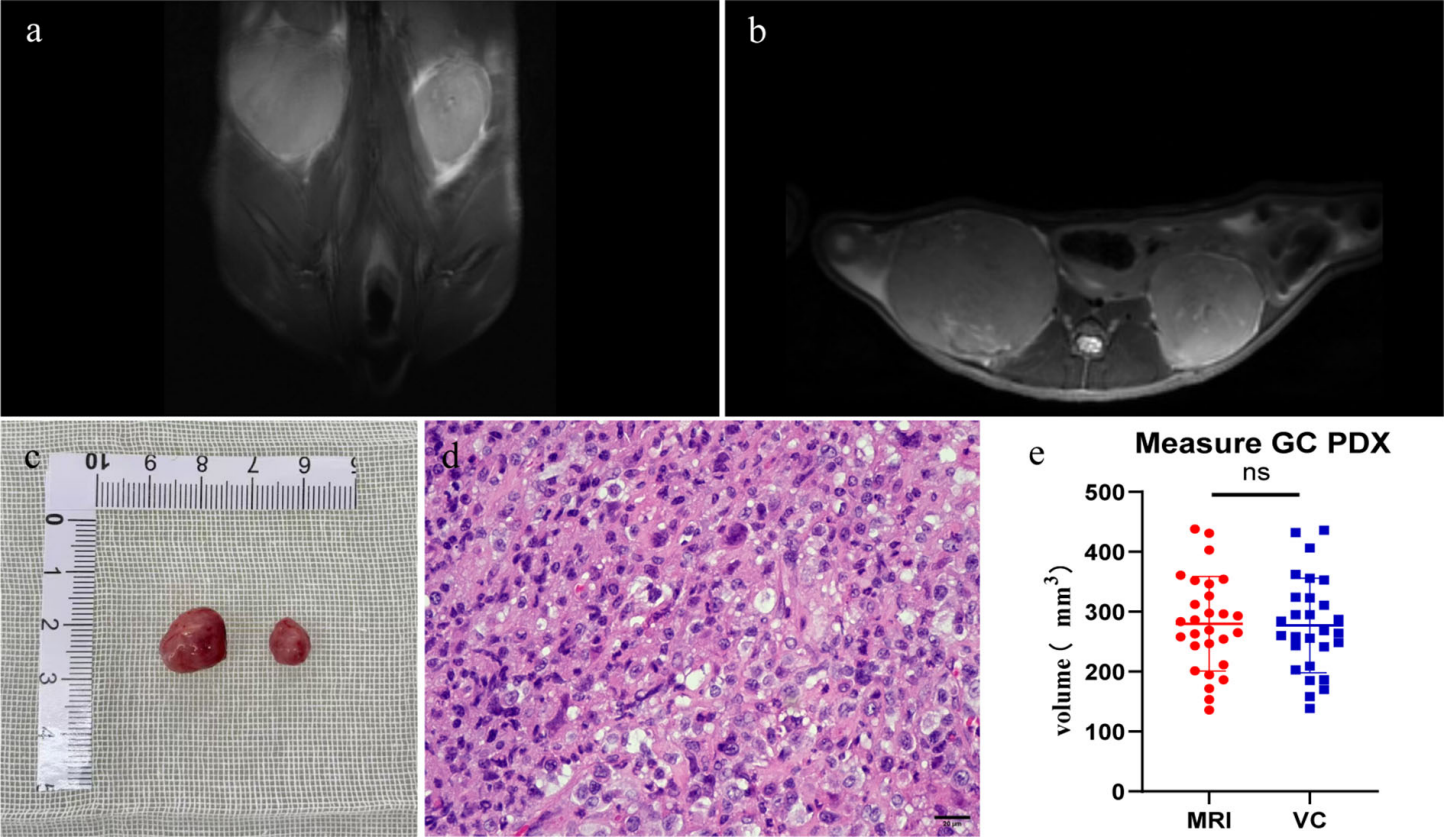
Comparison between MRI and vernier caliper (VC) measurements: Pre-excision MRI: T2WI sequences were performed **(a)** coronal plane; **(b)** transverse plane. Post-excision: **(c)** the PDX tumor was extracted. **(d)** histopathological analysis verified the tumor as gastric adenocarcinoma. **(e)** no significant difference was observed between tumor volumes calculated from MRI and VC measurements (ns, P > 0.05. n=28).

Weekly MRI observations were performed on the PDX. The tumor’s long and short diameters were measured using the software’s measurement tools. Serial observation of a PDX model revealed tumor growth dynamics, with measured long diameters of 2.5 mm, 2.1 mm, 2.0 mm, and 2.7 mm on days 8, 16, 25, and 32, respectively (**Figure 2**). The PDX tumor showed, Isointense and Hypointense on T1WI (**Figure 3a**), obvious Marked enhancement on T1WI contrast-enhanced(T1WI+C) scans (**Figure 3b**), Isointense and Hyperintense signal on T2WI (**Figure 3c**), companying with renal compression. When the tumor volume reached 100–500 mm³, the T2WI sequence was used to measure and calculate the tumor volume in the coronal (**Figure 4a**) and transverse (**Figure 4b**) planes. Subsequently, the mice were euthanized, and the tumors were excised (**Figure 4c**), Histopathological analysis diagnosed gastric adenocarcinoma (**Figure 4d**). The length and width of the tumors were measured using a vernier caliper, and the tumor volume was calculated. A comparison with the last MRI results showed no statistically significant difference between MRI and vernier caliper measurements of tumor volume: [(279.91 ± 78.94) vs. (277.10 ± 78.79), t = 1.41, P = 0.171] (**Figure 4e**).

### 3.5 Comparison of PDX and primary tumor consistency

**Figure 5:**
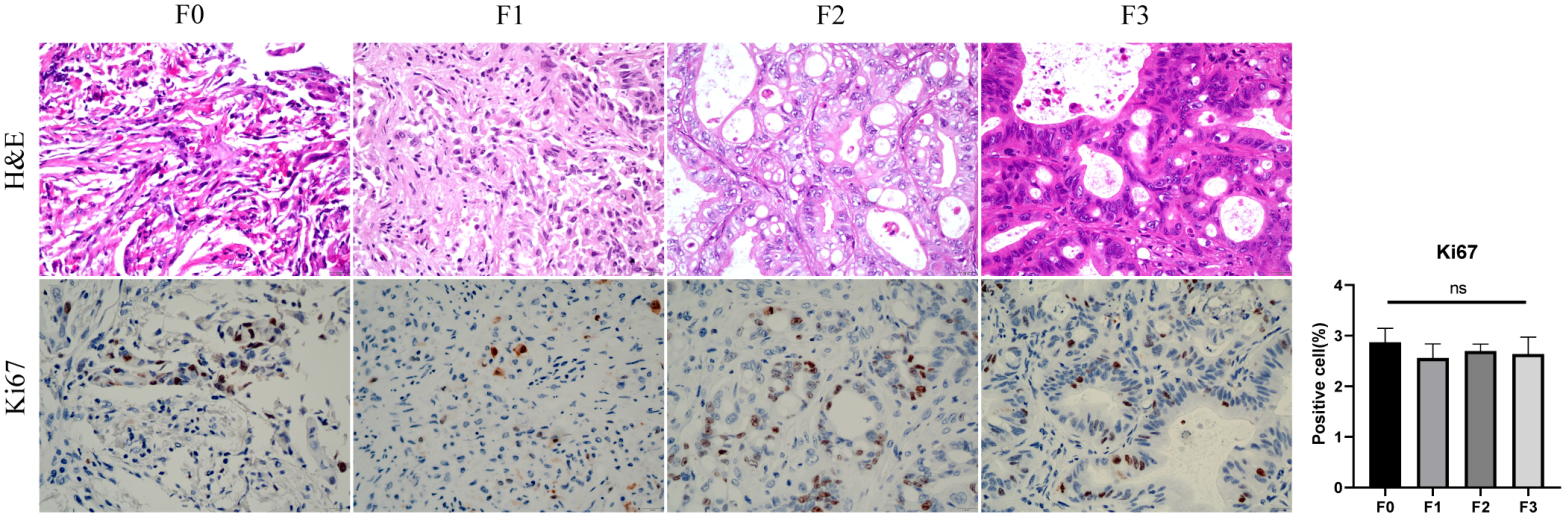
The comparison of HE and Ki67 between primary tumor(F0) and PDX(F1-3). F0, F1, F2 is poorly differentiated gastric adenocarcinoma, F3 is Moderate to poorly differentiated gastric adenocarcinoma, Ki67-positive cell (%) showed no difference between primary tumors and PDXs (ns, P > 0.05).

Taking out the tumor tissue in each generation of the PDX, performing H&E and IHC. F0, F1, F2 is poorly differentiated gastric adenocarcinoma, F3 is Moderate to poorly differentiated gastric adenocarcinoma, KI67 is expressed in primary tumor and PDXs. No statistically significant difference was observed in Ki67-positive cell counts among F0 through F3 generations (**Figure 5**).

## 4 Discussion

The PDX model serves as a valuable tool for investigating tumorigenesis mechanisms, developing personalized therapies, and evaluating drug efficacy (21). The US National Cancer Institute recommends prioritizing PDX models over conventional *in vitro* cell cultures for drug screening (22). However, gastric cancer PDX models present several challenges: (a) low success rates, (b) prolonged tumor growth periods that fail to meet clinical timelines, (c) unidentified factors contributing to extended growth duration, and (d) the absence of non-invasive, objective methods for monitoring PDX progression (23–25). Studies by Lee et al. (26) suggest improved engraftment rates with renal capsule implantation, this study specifically aims to identify critical factors influencing gastric cancer PDX transplantation in the renal capsule and explore optimal MRI sequence parameters for observing renal capsule PDX.

In this study, we successfully established 28 out of 73 PDX models (38.36%) and identified key parameters associated with engraftment success rates in gastric cancer PDX in the renal capsule. The success rate of engraftment is associated with the transplantation site. In the study by Ji et al., the engraftment success rate was 12% in the first-generation subcutaneous left thigh (19). In our study, the success rate achieved with renal capsule engraftment was 38.36%. In contrast, Yu et al. reported a success rate of 39.6% for subcutaneous right flank engraftment (20). Our findings demonstrate that ex vivo time and transplant tissue size are critical experimental factors affecting successful PDX establishment. While Choi et al. (27) reported optimal engraftment with ex vivo times under 75 minutes, our study achieved higher success rates when maintaining ex vivo time in 2 hours. As ex vivo duration directly impacts tissue ischemia and cellular viability, minimizing this interval is crucial for transplanting viable tumor cells. Successful tissue attachment at the implantation site is crucial for revascularization. Due to the limited physiological space in the renal capsule, 3×3×3 mm tissue fragments were occasionally expelled, whereas both 1×1×1 mm and 2×2×2 mm fragments maintained stable attachment. Furthermore, compared to 1×1×1 mm fragment, the 2×2×2 mm fragments contained a greater number of tumor cells. According to previous reports, among the clinicopathological factors investigated by Kuwata et al., pathological lymph node metastasis status showed a significant correlation with PDX establishment success rates (18). In our study, the engraftment success rate was not associated with age, gender, tumor pathological type, differentiation degree, AJCC stage, or Borrmann classification, which is consistent with findings from other studies (28, 29). Additionally, we found that patient serum albumin levels were related to PDX success rates. It’s indicators of good nutritional status in patients (30, 31), suggesting that tumor cells from well-nourished patients may have higher viability. This finding differs from the results reported by Choi et al. (27).

In PDX models, caliper measurements can be used to determine tumor size; however, this method proves challenging for gastric cancer PDX models in the renal capsule, typically requiring serial necropsies. Previous studies have found that among FDG-PET/CT, contrast-enhanced MRI and non-contrast MRI, non-contrast T2w MRI was the most effective and efficient imaging technique (32). Similarly, our study compared T2WI sequences with contrast-enhanced T1WI (T1WI+C) and found both modalities could clearly visualize renal capsule PDX. Notably, T2WI eliminated the need for contrast agent injection, thereby reducing examination time and avoiding potential trauma to mice from contrast administration. In this study, renal capsule PDX was first detected as early as day 8. On imaging, the PDX exhibited Hyperintense on T2WI and Hypointense intensity on T1WI compared to adjacent muscle tissue, with post-contrast marked enhancement indicating vascularization. These findings demonstrate MRI’s capability for early PDX detection. Consistent with prior research (33), our measurements from T2WI sequences showed no significant difference from caliper measurements, with pathological confirmation of gastric cancer PDX. Previous studies have demonstrated that PDX tumors maintain consistent histological characteristics compared to their original primary tumors (34–37). Our findings align with this observation, as the PDX models from F0 to F3 generations preserved the histological features of poorly differentiated adenocarcinoma. Notably, the number of Ki67-positive cells - reflecting cellular proliferative activity (38) - showed no significant differences across the F0-F3 generations.

In summary, our study demonstrates that for establishing gastric cancer PDX models in the renal capsule of NOD/SCID mice: (1) implantation of 2×2×2 mm tumor tissue within 2 hours post-excision represents the optimal experimental parameter for enhancing engraftment efficiency; (2) MRI enables early detection and longitudinal monitoring of PDX growth,T2WI sequence serves as an effective imaging modality for monitoring renal capsule PDX tumors; and (3) PDX models maintain histological fidelity with primary tumors. These findings provide an experimental foundation for translating PDX models into clinical practice and developing personalized therapeutic strategies for gastric cancer.

## Data Availability

The original contributions presented in the study are included in the article/**Supplementary Material**. Further inquiries can be directed to the corresponding author.
