# Supplementary material for: Renal capsule patient-derived xenograft model for gastric cancer: establishment and MRI characterization
Source: Front Immunol. 2025 Nov 5;16:1683916. doi: 10.3389/fimmu.2025.1683916 (PMC12631216; doi:10.3389/fimmu.2025.1683916)
Supplement: Supplementary file 3 [file Supplementaryfile1.docx]

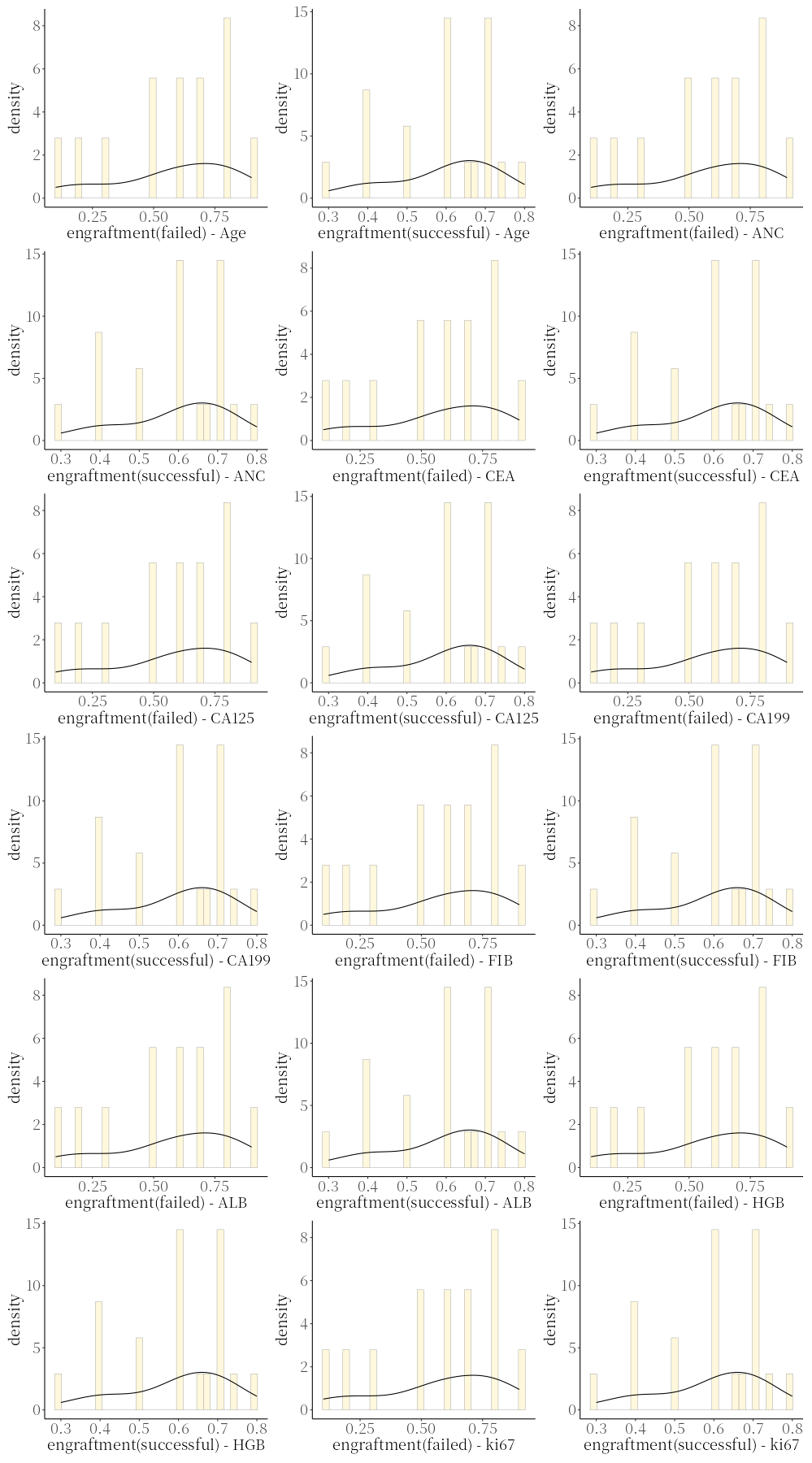


**Supplementary Figure 1:** Histogram of variables. Absolute Neutrophil Count (ANC), Hemoglobin (HGB), Fibrinogen (FIB), Carcinoembryonic Antigen (CEA), Carbohydrate Antigen 125 (CA125), Carbohydrate Antigen 19-9 (CA199), Albumin (ALB), Proliferation marker protein Ki-67 (Ki67), Age. All variables followed a normal distribution.
